# Comparison of weekly and single dose intraarticular recombinant human growth hormone injection on cartilage degeneration in osteoarthritic model of white New Zealand rabbits

**DOI:** 10.1186/s40634-022-00458-y

**Published:** 2022-02-21

**Authors:** Andri Maruli Tua Lubis, Mohammad Triadi Wijaya, Bambang Pontjo Priosoeryanto, Riky Febriansyah Saleh, Syahdi Farqani

**Affiliations:** 1 Department of Orthopaedic and Traumatology, Faculty of Medicine, Universitas Indonesia/Dr. Cipto Mangunkusumo National Hospital, Jakarta, Indonesia; 2 Department of Veterinary Pathology, Faculty of Agriculture, Institut Pertanian Bogor, Bogor, Indonesia

**Keywords:** Repeated growth hormone, Cartilage repair, Animal experiment

## Introduction

Osteoarthritis (OA) is a heterogeneous condition characterized by the defect of articular cartilage integrity accompanied by several pathological changes in all articular structures including the subchondral bone and synovium [2]. This condition is followed by related signs and symptoms such as pain and decrease in joint function. The global prevalence of knee osteoarthritis increases with age. It was 16% in population aged 15 and over and was 22.9% in population aged 40 and over [3]. The worldwide burden due to this musculoskeletal disorder also increased. As a leading cause of disability worldwide, OA has significantly increased the socioeconomic burden by 63.1% between 1990 and 2007 and by 31.4%between 2007 and 2019 [11].

Cartilage growth and metabolism from embryogenesis to adulthood are all regulated by certain specific hormonal factors. Growth hormone (GH) is known to be involved in various growth processes since it has systemic effect on anabolism throughout the body. It can stimulate cell growth, reproduction, and regeneration, including articular chondrocytes. The stimulatory effect of GH on articular chondrocytes can be directly or indirectly, mediated by insulin-like growth factor-1 (IGF-1) [19]. Intraarticular growth hormone has also a local effect on the subchondral bone by inducing a modified angiogenesis which is called morphoangiogenesis. Morphoangiogenesis process induced by GH can produce structures containing histiocytes and stem cells. The stem cells produced in morphoangiogenesis process are capable to regenerate the articular cartilage [5]. A study by Kim et al. also showed a positive effect on articular cartilage of the rabbit with osteoarthritis that was injected with intraarticular GH [10]. Recent study has also shown positive results of intraarticular growth hormone injection compared with hyaluronic acid or placebo [12]. On advanced osteoarthritis of the knee, cartilage destruction is usually severe and involved more than one compartment of the knee. The positive effect of GH on cartilage regeneration has been reported in in vitro and animal studies as well as in human early and advanced OA studies [1, 5, 8, 10, 12, 14, 17–19]. However, intraarticular GH injection has not yet been implemented as a standard treatment option since there lacks a standard dose for the knee osteoarthritis.

The effect of growth hormone on chondrocyte proliferation could be directly or mediated by insulin-like growth factor-1 (IGF-1) [4, 19]. In an in vitro study, stimulation of DNA synthesis of articular chondrocytes by GH was dose-dependent [19]. Therefore, to promote cartilage regeneration in severe OA, maintaining GH level in the synovial fluid will be necessary. Ok et al*.* found that twice weekly dose of local GH injection lowered osteoarthritic scores in the cartilage and subchondral bone of temporomandibular joint osteoarthritis in rat model [14]. However, there is no dose ranging study of intraarticular GH injection to determine the effective dosage for cartilage defect of the knee. In this study, we evaluated whether maintaining GH levels by repeated GH intra-articular injection would result in better cartilage regeneration compared to single intra-articular GH injection.

## Materials and methods

This animal study was conducted in Educational Animal Hospital, Bogor, Indonesia. All procedures performed in this experimental study were in accordance with the ethical standards and approved by the Ethical Committee of Educational Animal Hospital, Faculty of Veterinary Medicine (Ethical Approval Number: 078/KEH/SKE/1), Institut Pertanian Bogor, Indonesia. This study has been reported in line with the ARRIVE guidelines [16].

### Experimental animals

Twenty-four male, skeletally mature New Zealand white rabbits, aged 8–9 months, weighed 1800–2500 g were included as models for knee osteoarthritis in this experimental study. All rabbits were caged separately and acclimatized for 10 days at an animal hospital. Room temperature, air humidity, and lighting were adjusted daily. All subjects received bedding, fresh water, and nutritionally balanced food. They were screened for any disease and confirmed healthy (no infection, no congenital disorder, and no history of trauma in the lower extremity) by a veterinarian. Blinding was applied during preparation of the injected drugs, process of injection, and while measuring evaluated variables.

### Osteoarthritis induction procedure

Two milligrams of type II collagenase extracted from *Clostridium histolyticum* (Sigma-Aldrich, St. Louis, MO, USA) was injected to the knee joint cavity of the experimental animals. This proteolytic enzyme contains active proteinase that degrades collagen and proteoglycan of articular cartilage. The dose was repeated after 3 days. Degeneration effects of collagenase to the cartilage were expected after 2 weeks post-injection [9]. We have done a preliminary study of that same osteoarthritis induction procedure into three other rabbits, not the study subjects, and confirmed macroscopically and histologically the degeneration effect of type II collagenase.

### Experimental design

Two weeks after induction, subjects were randomly divided using Federer’s formula into four groups (*n* = 6 per group) with four different treatments. The control group was treated with weekly intra-articular placebo injection of 1 mL normal saline (NaCl 0.9%) for five weeks. Based on previous study, the growth hormone (GH) groups received intraarticular injections of human recombinant growth hormone (Novell-Eutropin™, Novell Pharmaceutical) at the dose of 2 mg (4 IU) [10, 12]. The GH1 group received one time of the intraarticular injections of human recombinant growth hormone, followed by placebo injection weekly from week 2 until week 5. Group GH3 received weekly intraarticular injections of human recombinant growth hormone for 3 weeks, followed by placebo injection for week 4 and 5. The GH5 group received weekly human recombinant growth hormone intra-articular injection for 5 weeks. Both GH and placebo injections were prepared in same type of Terumo® syringes by a veterinarian at the Educational Animal Hospital, Faculty of Veterinary Medicine, Institut Pertanian Bogor, who was not involved in the administration of the study drugs.

### Macroscopic and histopathologic assessments of osteoarthritis

All subjects were monitored for eight weeks after the first injection, weighed regularly. At the end of week 8, all subjects were euthanized by 10 mg phenobarbital intravenous injection. The knee joints and it surrounding tissues were dissected. Specimens for histopathological evaluation were taken in sagittal slices from the posterior lateral condyle of the rabbit’s femur, fixed in 10% formalin buffer, decalcified using 20% EDTA, and cut in a coronal angle to obtain two or three sections with five-micrometer thickness. Specimens were stained using Hematoxylin and Eosin (HE) [10].

Macroscopic and microscopic (histopathologic) scoring systems were described in Table 1. Macroscopic scoring criteria by Yoshimi et al. was used to evaluate articular cartilage destruction of knee joint [21]. Degree of microscopic cartilage destruction was measured by using modified Mankin scoring criteria [20].

**Table 1:** Scoring systems for macroscopic and microscopic evaluation

Mankin Microscopic Scoring System	Value	Yoshimi Macroscopic Scoring System	Value
**Structure**			
Normal	0	Normal cartilage	0
Irregular surface	1	Softening	1
Pannus and irregular surface	2	Fibrillation	2
Cleft through transitional zone	3	Erosion	3
Cleft through radial zone	4	Ulceration	4
Cleft through calcification zone	5	Cartilage damage	5
Complete disorganization	6		
**Cell**			
Normal	0		
Diffuse hypercellularity	1		
Cloning	2		
Hypocellularity	3		
**Total score**		**Total score**	
Minimum score	0	Minimum score	0
Maximum score	9	Maximum score	5

All assessments of each specimen were performed by two blinded certified veterinary pathologists to reduce potential bias.

### Statistical analysis

Data were tested by using ANOVA parametric test and Mann–Whitney non-parametric test. All data obtained were analyzed by using Statistic Program for Social Science (SPSS) version 20.0 for Windows (Chicago, IL). The level of significance was considered at *P value* less than 0.05.

## Results

All subjects were survived at the end of treatment observation, thus all measurements and treatment were conducted as per protocol. The baseline weights of all rabbits were comparable (*P value* 0.901). The weight gain was observed in all rabbits during the study, and there was no significant difference in the mean weight increase among all groups (*P value* 0.767).

### Preliminary study

The preliminary study showed destruction of the cartilage mimicking cartilage changes in osteoarthritic joint. The cartilage was softened with a Yoshimi score of 3, while histopathological appearance showed cloning and tearing that reached the transitional zone of the cartilage which was equal to 5 points on the Mankin score.

### Macroscopic assessment

**Fig. 1:**
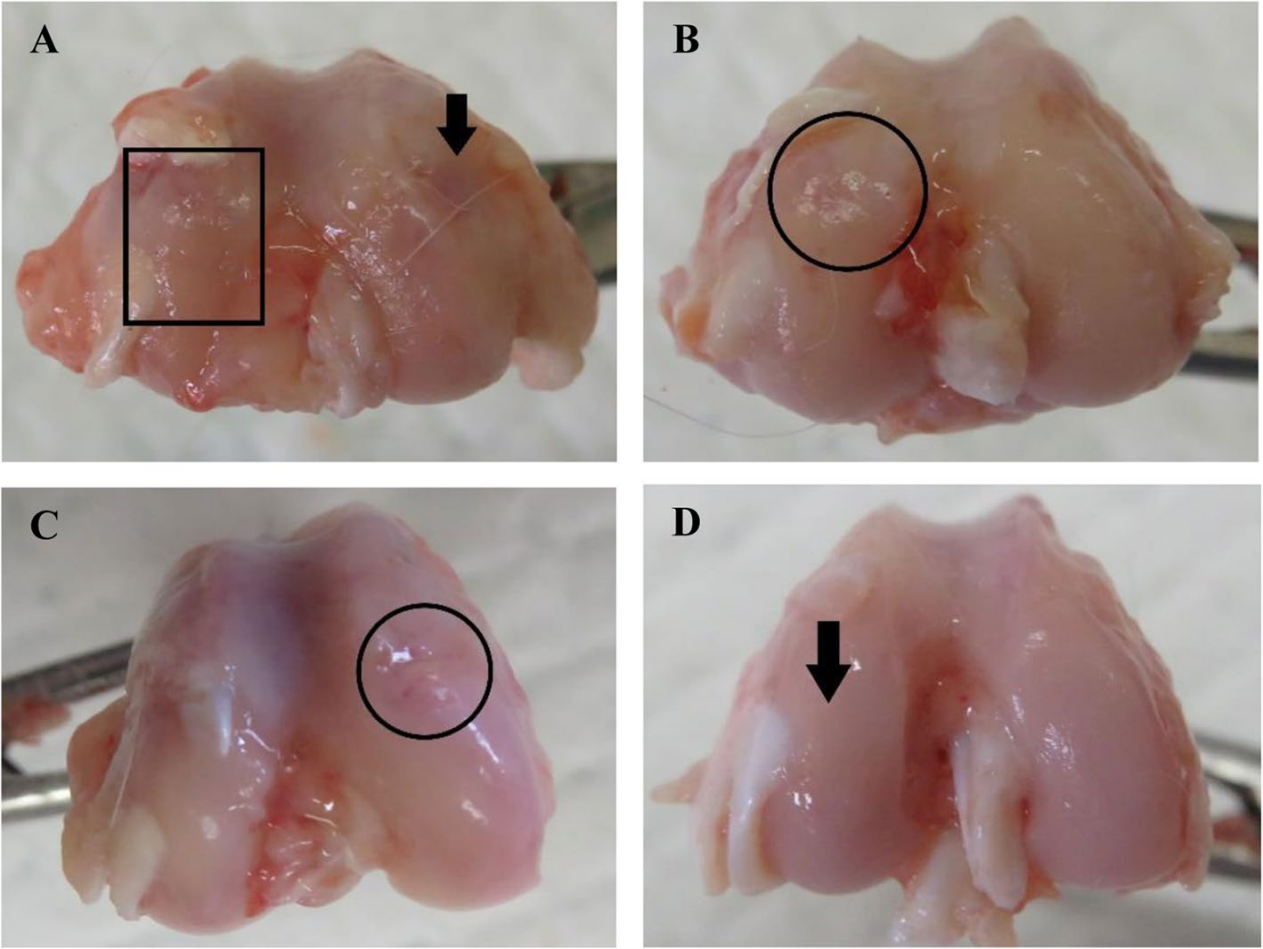
Macroscopic appearance of the cartilage. **A** Ulceration and erosions on cartilage in control group. **B** Cartilage fibrillation in GH1 group. **C** Cartilage fibrillation in GH3 group. **D** Near-normal cartilage in GH5 group

The changes of macroscopic appearance on articular cartilage after treatment were assessed by Yoshimi scoring system. The normal distribution of Yoshimi score was found in control group (*P value* > 0.05) while that score distribution in the other groups were non-normal (*P value* < 0.05) (Table 2). Different macroscopic appearances between groups of treatments were observed. Ulceration and erosion were found on cartilage in control group (Fig. 1A). Cartilage fibrillations were found in GH1 and GH3 groups (Fig. 1B and C, respectively), whereas the cartilage surface in GH5 group was smooth with focal softened cartilage but no fibrillation (Fig. 1D).

**Table 2:** Yoshimi score distribution

	**Score**	***P* value**
Control	3.17 (0.75)	0.212
GH1 group	2.00 (2.0 – 4.0)	0.006
GH3 group	1.50 (1.0 – 2.0)	0.004
GH5 group	1.00 (0 – 1.0)	0.001

*GH1* Single dose growth hormone injection, *GH3* 3 times weekly dose growth hormone injection, *GH5* 5 times weekly dose growth

Comparative *post-hoc* analysis showed a statistically significant improvement of Yoshimi scores between GH3 group compared to control group and GH5 group compared to control group with *P value* 0.004 and 0.002, respectively. The improvement of Yoshimi score in GH5 group was significantly greater than the improvement score of GH1 group with *P* = 0.002 as presented in Table 3.

**Table 3:** Statistical analysis and comparison of Yoshimi score between groups

Yoshimi Macroscopic Score	*P* value
Control *vs.* GH1 group	0.180
Control *vs.* GH3 group	0.004*
Control *vs.* GH5 group	0.002*
GH1 *vs.* GH3	0.065
GH1 *vs.* GH5	0.002*
GH3 *vs.* GH5	0.065

*p<0.005; Post−hoc test analysis; *GH1*, single dose growth hormone injection, *GH3* 3 times weekly dose growth hormone injection, *GH5* 5 times weekly dose growth hormone injection, Control: placebo

**Fig. 2:**
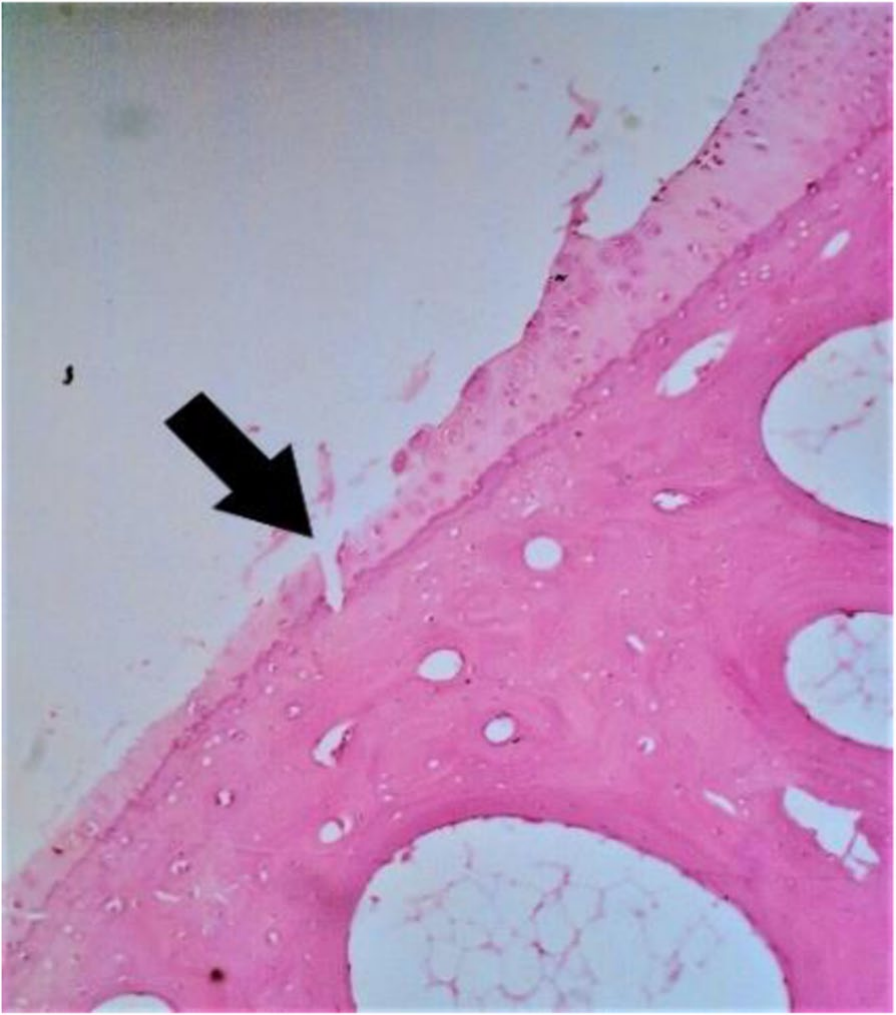
Microscopic appearance in control group showing tear through the tidemark and large hypocellular area (arrow)

**Fig. 3:**
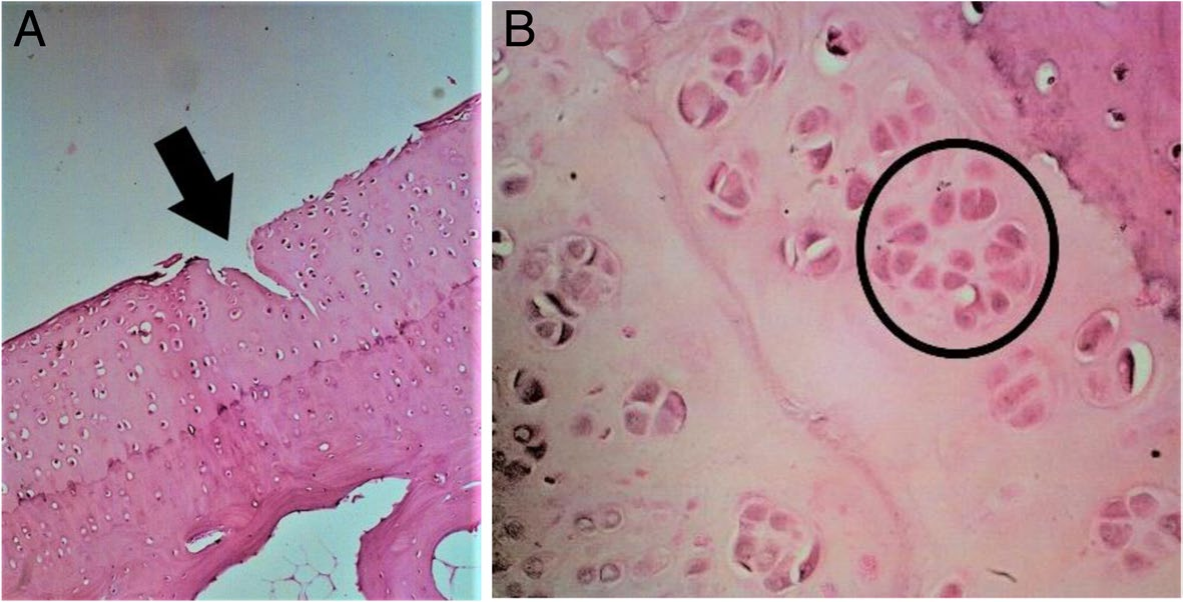
Microscopic appearance in GH1 group. **A** Cartilage tear reaching the transitional zone (arrow). **B** Cell cloning (circle) along with irregular surface of cartilage

**Fig. 4:**
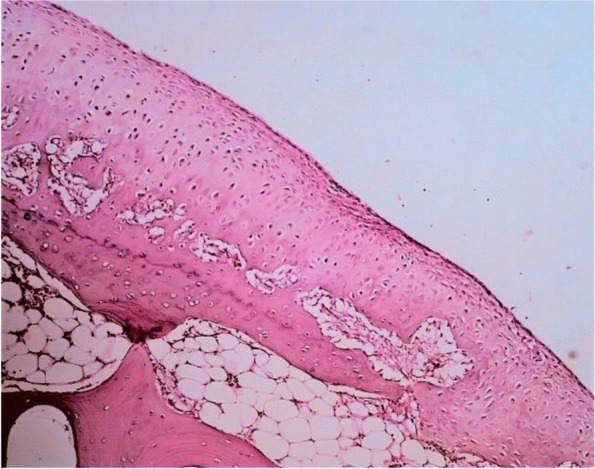
Microscopic appearance in GH3 group showing hypercellular area of cartilage

**Fig. 5:**
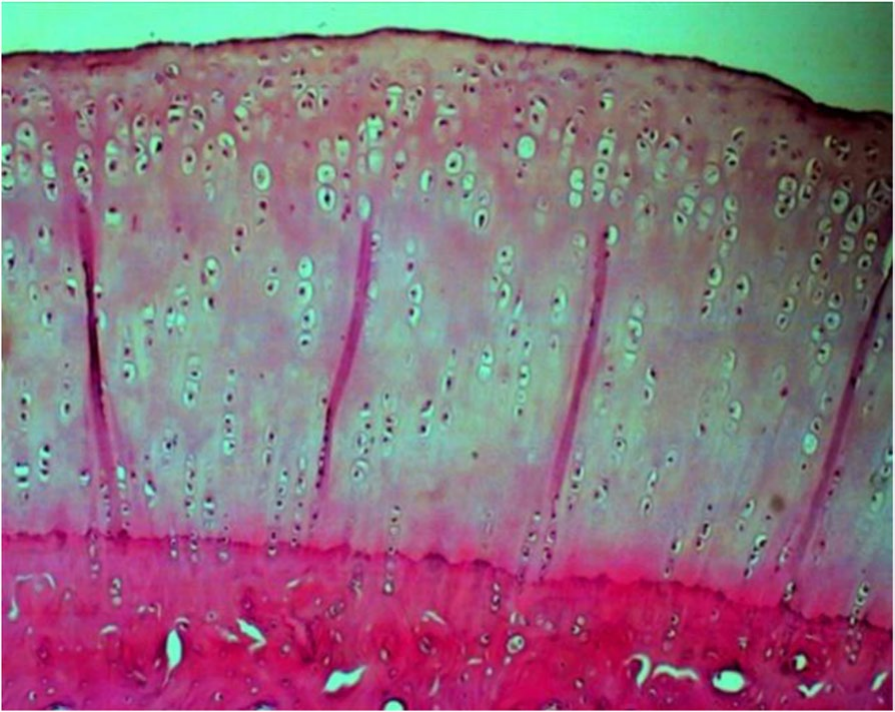
Microscopic appearance in GH5 group showing normal and thicker cartilage

Histopathological assessment was performed by determining the Mankin score. A massive tear through the tidemark and large hypocellular area were seen in the control cartilage in Fig. 2. While in GH1 group, cartilage tear only reached the transitional zone (Fig. 3A) and chondrocytes cloning as well as irregular surface of cartilage (Fig. 3B) was observed. On the contrary, no cartilage tears found on the GH3 and GH5 groups. However, irregular cartilage surface and hypercellular area of cartilage were still detected on the GH3 group (Fig. 4). Whereas in GH5 group, normal cartilage surface as well as normal cellularity were observed as a thick cartilage (Fig. 5).

Statistical analysis of Mankin scores showed normal distribution in each group and comparative test resulted in a significant difference between each group (*P value* < 0.001). *Post-hoc* test revealed that only the comparison of Mankin score between GH1 and GH3 groups did not show significant difference (Table 4).

**Table 4:** Statistical analysis and comparison of Mankin score between groups

Mankin Microscopic Score	Mean Difference	CI 95%		*P value*
		**Min**	**Max**	
**Control *vs.* GH1 group**	3.17	1.06	5.27	0.004*
**Control *vs.* GH3 group**	4.17	2.32	6.01	< 0.001*
**Control *vs.* GH5 group**	5.50	3.65	7.34	< 0.001*
**GH1 *vs.* GH3**	1.00	- 0.79	2.79	0.352
**GH1 *vs.* GH5**	2.33	0.54	4.12	0.013*
**GH3 *vs.* GH5**	1.33	0.003	2.66	0.049*

*p<0.005; Post hoc Games−Howell test; *GH1* single dose growth hormone injection, *GH3* 3 times weekly dose growth hormone injection, *GH5* 5 times weekly dose growth hormone injection, Control: placebo

## Discussion

Growth hormone has not yet been indicated for therapy in osteoarthritis although it has the capability to repair cartilage defect when injected intraarticularly [10, 12]. In an animal study, Dunn et al. has injected 3.75 IU of growth hormone into the arthritic joint and resulted in a morphoangiogenesis as a cartilage response from intraarticular injection of GH [5]. However, there is no evidence yet whether the repeated dosage of exogenous growth hormone has effect on the cartilage regeneration. This current study aimed to investigate the effect of repeated GH intra-articular injection compared to single GH intra-articular injection on repairing chondral defects of articular cartilage of the knee in rabbits. Based on previous study, four IU of growth hormone per intra-articular injection was used for three weeks [12]. In this study, the same weekly dose of GH was applied for a week in the GH1 group, for three weeks in the GH3 group and for five weeks in GH5 group. The longer duration of weekly GH injection has resulted in a greater improvement compared with the shorter duration ones. These findings suggested that maintaining the GH levels in the synovial fluids might lead to a continuous stimulation of IGF-1, which in turn will stimulate chondrocyte proliferation and type II collagen synthesis and keep anabolic phase at an optimal condition [9, 13]. In accordance with this study, Dunn et al. has also injected repeated weekly GH dose in 14 arthritic ankles and found clinical improvement as demonstrated by increased range of motion and visual analog scale (VAS) score [6].

The microscopic and macroscopic assessments of cartilage are relevant to quantify the level of damage caused by osteoarthritis degeneration process, as well as to observe the healing process. Relevant and applicable scoring systems were conducted in this study. Many studies on osteoarthritic animal model also evaluated the microscopic changes by using Mankin scoring system. The Mankin score ranged from 0 to 9 reflecting pathological changes in articular cartilage, with 0 for normal condition and score 9 indicating complete disorganization along with hypocellular condition [10, 12, 18]. Ostergraad et al. reported that Mankin maximum score described significant articular changes, as shown in severe osteoarthritis condition. This scoring system has wide inter-observer and intra-observer variation although it is easy to use and has a validated scoring correlation with histochemical changes in OA model [15]. Macroscopic evaluation using Yoshimi score is used to determine the level of cartilage destruction [21]. To overcome the inter-observer and intra-observer variation in Mankin scoring system, the macroscopic scoring should be included in combination with this microscopic scoring to provide a more reliable and trustable result [15]. In addition to that, to reduce potential bias in the assessment of both microscopic and macroscopic appearances was performed by two blinded certified veterinary pathologists.

In accordance with the result of intraarticular GH injection in this study, several studies have reported the effect of GH to the regeneration of articular cartilage. Tzukazaki et.al found that the regeneration effect of GH is induced by two mechanisms. The GH will stimulate chondrocytes by inducing the somatomedin C at both protein and mRNA level and GH itself has direct proliferative effect that proved by the expression of the proto-oncogene c-myc after GH administration [19]. A study by Rahimdazeh et al. showed better Western Ontario and McMaster Universities Osteoarthritis Index (WOMAC) Score evaluated from human knee injected with platelet rich plasma (PRP) combined with four IU growth hormones when compared with PRP only [17].

Macroscopic and microscopic assessment in this study shows significant higher Yoshimi and Mankin scores in groups receiving weekly repeated dose than those in the control group. Kim et al. and our previous study found similar positive results of intraarticular GH injection, showing a significantly better healing either in combination with hyaluronic acid or when given as single agent [10, 12]. Improvement of Mankin score in GH5 group, showing thicker cartilage and more organized cellular structure, is strong evidence that GH plays a role in cartilage repair. Another study in osteoarthritic horse model revealed that cartilage and subchondral repair was faster in the group receiving somatomedin C, which is also known as insulin-like growth factor-1 (IGF-1), a protein that is stimulated by GH [7]. This phenomenon was also found by Tsukazaki et al. where GH stimulated somatomedin C in RNA level and has a direct effect to specific protein in chondrocyte [19].

There are some limitations of this study. The duration of repeated intraarticular GH injection was up to five weeks so that the optimum frequency of the GH injection still could not be determined in this study. Due to limitation of time and facility, this study lacks immunohistochemistry assessment and DNA detection test to identify collagen synthesis as the effect of GH injection on cartilage repair. However, the result of this study showed a potential benefit of repeating GH injection for cartilage healing. Further study could be performed in OA patients to determine the optimum cycle of intraarticular GH injection by evaluating clinical and biomarker parameters.

This study was conducted to evaluate whether increasing the cycle of the intraarticular GH injection will result in a better cartilage healing as assessed by Yoshimi and Mankin scoring system. The comparison of Yoshimi score between GH3 and GH5 groups showed no significant difference but the Mankin score of GH5 group was significantly better than that of the GH3 group. Therefore, in this study the five-cycle GH injection was the optimum dosage since it almost reached a plateau.

## Conclusion

Repeated doses of intraarticular injection of GH give better result compared to single dose and control group. Five weeks intraarticular GH injection was the optimum dose among the other dosages. Further research should be performed by using biomarker of cartilage growth and more cycle to specify the optimum cycle of GH intra-articular injection for osteoarthritis patient.

## Abbreviations

OA: Osteoarthritis
GH: Growth hormone
IGF-1: Insulin—like growth factor-1
EDTA: Ethylenediamine tetra acetic acid
HE: Hematoxylin and eosin
ANOVA: Analysis of variance
SPSS: Statistical Package for the Social Sciences
WOMAC: The Western Ontario and McMaster Universities Osteoarthritis Index
PRP: Platelet rich plasma
RNA: Ribonucleic acid
